# A sticky situation: the unexpected stability of malaria elimination

**DOI:** 10.1098/rstb.2012.0145

**Published:** 2013-08-05

**Authors:** David L. Smith, Justin M. Cohen, Christinah Chiyaka, Geoffrey Johnston, Peter W. Gething, Roly Gosling, Caroline O. Buckee, Ramanan Laxminarayan, Simon I. Hay, Andrew J. Tatem

**Affiliations:** 1 Department of Epidemiology, Johns Hopkins Bloomberg School of Public Health, Baltimore, MD, USA; 2 Malaria Research Institute, Johns Hopkins Bloomberg School of Public Health, Baltimore, MD, USA; 3 Emerging Pathogens Institute, University of Florida, Gainesville, FL, USA; 4 Center for Disease Dynamics, Economics and Policy, Washington, DC, USA; 5 Fogarty International Center, NIH, Bethesda, MD, USA; 6 Clinton Health Access Initiative, Boston, MA, USA; 7 Spatial Ecology, Department of Zoology, University of Oxford, Oxford, UK; 8 Malaria Elimination Initiative, Global Health Group, University of California San Francisco, San Francisco, CA, USA; 9 Center for Communicable Disease Dynamics, Department of Epidemiology, Harvard School of Public Health, Boston, MA, USA; 10 Department of Geography and Environment, University of Southampton, Highfield, Southampton, UK

**Keywords:** malaria elimination, malaria eradication, backwards bifurcation

## Abstract

Malaria eradication involves eliminating malaria from every country where transmission occurs. Current theory suggests that the post-elimination challenges of remaining malaria-free by stopping transmission from imported malaria will have onerous operational and financial requirements. Although resurgent malaria has occurred in a majority of countries that tried but failed to eliminate malaria, a review of resurgence in countries that successfully eliminated finds only four such failures out of 50 successful programmes. Data documenting malaria importation and onwards transmission in these countries suggests malaria transmission potential has declined by more than 50-fold (i.e. more than 98%) since before elimination. These outcomes suggest that elimination is a surprisingly stable state. Elimination's ‘stickiness’ must be explained either by eliminating countries starting off qualitatively different from non-eliminating countries or becoming different once elimination was achieved. Countries that successfully eliminated were wealthier and had lower baseline endemicity than those that were unsuccessful, but our analysis shows that those same variables were at best incomplete predictors of the patterns of resurgence. Stability is reinforced by the loss of immunity to disease and by the health system's increasing capacity to control malaria transmission after elimination through routine treatment of cases with antimalarial drugs supplemented by malaria outbreak control. Human travel patterns reinforce these patterns; as malaria recedes, fewer people carry malaria from remote endemic areas to remote areas where transmission potential remains high. Establishment of an international resource with backup capacity to control large outbreaks can make elimination stickier, increase the incentives for countries to eliminate, and ensure steady progress towards global eradication. Although available evidence supports malaria elimination's stickiness at moderate-to-low transmission in areas with well-developed health systems, it is not yet clear if such patterns will hold in all areas. The sticky endpoint changes the projected costs of maintaining elimination and makes it substantially more attractive for countries acting alone, and it makes spatially progressive elimination a sensible strategy for a malaria eradication endgame.

## 1. Introduction

Malaria was the first human disease formally scheduled for global eradication, when a vote of the 8th World Health Congress in 1955 made it the official policy and launched the Global Malaria Eradication Programme (GMEP), so malaria has a special place in the history of human disease eradication. By 1969, however, the GMEP had collapsed and the World Health Organization changed the malaria agenda for countries from imminent elimination to indefinite control [1,2]. Malaria was then neglected for decades. Bill and Melinda Gates' call for eradication [3] has reoriented strategic thinking about malaria since 2007, and global eradication was reinstated as the long-term policy goal by a consensus decision of the Roll Back Malaria Partnership (RBMP) in 2008 [4,5]. Decisions about elimination are taken at the national level, with advice given to formally assess the feasibility of malaria elimination [6]. Zanzibar conducted the first (and still only) malaria elimination feasibility assessment in 2009 [7]. Meanwhile, of the 99 countries that still have endemic malaria, 36 have already made plans to eliminate malaria [8]. The basis for making appropriate decisions about elimination policy and practice remains one of the most important research topics in malaria [5,9,10].

Malaria elimination involves stopping transmission in a defined region until no parasites remain [11,12]; eradication is elimination on a global scale. Elimination is currently guided by a theory of malaria transmission dynamics and control based largely on work by Macdonald [13–15]. The potential intensity of transmission under baseline conditions is described by the basic reproductive number, *R*_0_, the expected number of new human malaria cases arising from a single human malaria case [16]. Baseline entomological determinants of transmission are described by vectorial capacity in a population with no vector control, *V*_0_, and epidemiological aspects of baseline transmission are described by the net infectiousness of a human case with no immunity and no antimalarial drug use, *D*_0_, so that *R*_0 * *_is proportional to *V*_0_*D*_0_. Elimination must be achieved by interrupting transmission through implementation of vector control, treatment of infected individuals and usage of other available interventions, such as larval source management or active case detection. Vector control changes vectorial capacity to *V*_C_, and drugs and immunity change net infectiousness of human infections to *D*_C,I_. Transmission intensity in populations with some immunity and malaria control is described by the family of reproductive numbers (i.e. *R*_C,I_ is proportional to *V*_C_*D*_C,I_). Eliminating malaria on practical timelines, accounting for waning immunity, requires reducing transmission such that *R*_C,I_<0.5 [17]. After elimination, malaria importation is assumed to pose a constant threat because mosquito populations are expected to retain their potential to transmit malaria: adult vector control and larviciding change *V*_C_, but not *V*_0_. A particularly important number for planning is *R*_C_ (or alternatively, *R*_C,0_), which describes transmission under control without the effects of immunity. If control measures are insufficient (i.e. if *R*_C_ > 1), the reintroduction of malaria infections could lead to a return to endemic transmission once immunity wanes, and lowering *R*_C_ such that it is very close to zero shortens the expected length of transmission chains from each imported case [11]. Countries are therefore advised to retain the capability to control imported malaria until after eradication has been achieved, either by sustaining control measures even after elimination or through intense surveillance to identify and cure all imported and subsequent infections before they can lead to the resumption of transmission [18,19]. These continued measures make even the medium-term projected costs of elimination higher than maintaining existing control programmes [20]. Failure to sustain elimination following loss of immunity would leave populations vulnerable and raise the risk of a deadly resurgent malaria epidemic [21]. Elimination is thus regarded as a risky and, often, unwise strategy [22].

A major long-term issue for malaria elimination planning is thus the risk posed by malaria importation and how it can be managed. In Zanzibar, for example, the feasibility assessment showed that importation rates were the primary determinant of the cost and overall feasibility of sustainable elimination [7]. Many countries have insufficient capacity to organize the long-term operations needed to maintain malaria elimination, insufficient funds or competing priorities [23]. The GMEP had assumed that malaria elimination would be coordinated globally and, consequently, post-elimination planning for malaria importation was not prioritized since most countries could assume that their neighbours would also soon be eliminating malaria [12]. Without a campaign, some coordination is occurring through bilateral agreements or regional initiatives [24,25], but these efforts remain limited. The need to mitigate ongoing risks of malaria importation is, perhaps, the most basic difference between the endgame of a globally coordinated malaria eradication campaign and that of national/regional elimination plans based on decisions of individual countries.

Strategic decisions facing countries involve whether to eliminate malaria or minimize the burden through malaria control. Minimizing burden and eliminating malaria both involve sharply reducing transmission, but discussions about elimination and eradication are often seen as being in conflict with the ongoing efforts to minimize the burden of malaria, particularly in sub-Saharan Africa [22,26]. The rising tide of funding for malaria control has been stemmed by the global economic recession that started in late 2008. In the short-term, outside donor funding for malaria is expected to plateau at around 1.5 billion US dollars per year, far short of the amounts required to eradicate malaria [27]. With inadequate funding, it becomes more important than ever to prioritize interventions to reduce burden at low cost. In the current inadequate and uncertain funding environment, is it worth attempting elimination if loss of funding would inevitably lead to the unraveling of progress? The commitment to eradication and the recent history of malaria control have thus raised important questions about long-term planning for malaria [28].

Doubts persist about the possibility and practicality of interrupting transmission everywhere, as well as concerns about the costs, sustainability and risks of malaria elimination on the road to eradication. The questions are among the most important ones in malaria today, and they must be addressed with the best available science. The study of malaria eradication is, at least in part, a historical science that relies on case studies and retrospective analysis. The purpose of this article is to examine quantitatively some puzzling patterns evident in the history of malaria transmission and control and to reconsider what the observations and analysis suggest about the feasibility and desirability of pursuing malaria elimination and eradication.

## 2. A brief history of malaria eradication

Since 1900, and in particular since the end of World War II (WWII), the geographical range of malaria has contracted sharply [29,30]. The study of this contraction provides the richest source of information to inform the debate about elimination. Most of the contraction was caused either by GMEP activities or by some other factor (e.g. economic development) occurring at the same time as the GMEP that produced large and rapid reductions in transmission. The GMEP itself represented a major change in the approach to tackling malaria. Several important precursors to the GMEP were Soper's successful eradication of *Anopheles gambiae* s.l. from Brazil [31], discovery of the insecticidal properties of DDT, use of DDT by the military during WWII, successful field application of DDT-based indoor residual spraying (IRS) to control malaria [32], mass production of chloroquine and the formation of the World Health Organization [2]. After WWII, enthusiasm for a major global public health programme burgeoned and provided the momentum that launched the GMEP.

Though the GMEP officially started in 1955, activities had been expanding from the end of WWII [1]. Under the GMEP, DDT-based spraying programmes expanded in Europe, South America, Asia and the Pacific Islands [1]. GMEPs were more limited in Africa, however, because of concerns about the operational and technical challenges [33]. The GMEP was organized as a centrally managed programme [2,12], inspired largely by the military efficiency of the organization that allowed Soper to succeed in Brazil [31]. As the GMEP rolled out, the early phases involved large financial and programmatic investments and sharp declines in both transmission and burden. These were followed by long protracted periods with high costs, but without additional large declines in burden as countries worked to find and eliminate the last few cases. By 1969, the financing for the GMEP had collapsed, and the WHO had redefined its mission, generated new advice about malaria control, and released most of the GMEP advisory staff. The end of the GMEP saw large declines in spending on malaria and, at this point, countries found themselves at various points along the spectrum of progress towards elimination. The fates of these countries then diverged, largely as a function of how close they were to elimination. With the sharp declines in funding, many countries that reduced but did not eliminate malaria experienced resurgent malaria [1], while others had already eliminated malaria.

Feachem *et al.* [34] identified 131 attempted elimination programmes and classified them as `under way’, `successful’ or `unsuccessful’. Excluding the 32 countries where elimination was categorized as under way leaves 50 successful and 49 unsuccessful programmes. A recent systematic review documented 75 episodes of resurgent malaria in 61 countries [35] and can be used to examine the sustainability of these programmes. Of the 49 elimination programmes that failed to achieve elimination, resurgence events were identified in 36 (73%) of these countries. The causes of resurgence were poorly documented, but most frequently resurgence was blamed on the failure to sustain high intervention coverage levels. Resurgence is, in fact, the outcome generally predicted by the Ross–Macdonald theory of elimination, since the vector populations are expected to have retained their capacity to transmit malaria parasites. The fact that a majority of programmes saw eventual resurgence is, therefore, unsurprising within this conceptual framework.

What is surprising, however, is that of the 50 elimination programmes identified as successful, only four (8%) were found to have experienced resurgence, despite continued importation over many years. Moreover of these four, two eliminated malaria and are malaria-free once again. These 50 countries still have competent vectors and few have ongoing transmission-lowering activities. Such patterns do not appear to fit with accepted theory and thus merit closer examination.

**Figure 1.**
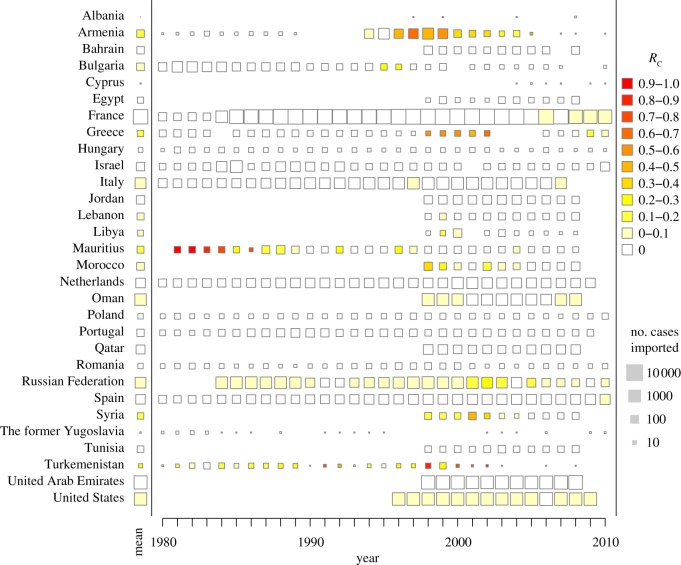
For 30 countries where data were available, data on imported malaria and any locally acquired malaria case were analysed to estimate the reproductive numbers for malaria, *R*_C_, under post-elimination levels of control in each year. The results are shown as the colour of the square. The average for all years is shown on the left, by the name of the country. The size of the square scales with the logarithm of the number of cases imported.

A recent analysis adds a new quantitative dimension to the problem [36]. Data on imported malaria were sought from the list of all elimination countries. Data were found from 30 countries, and 249 250 imported malaria cases were identified, compared with 4993 that were either ‘introduced’ cases (i.e. traced back to an imported case), or otherwise ‘locally acquired’. Using formulae derived from branching-process models [11], these data were analysed to estimate the reproductive numbers under control in these non-immune populations, denoted *R*_C,0_ (for details, see [36]). This analysis shows that estimated yearly reproductive numbers in elimination countries were (on average) approximately *R*_C,0_ ≈ 0.04, or approximately one locally acquired malaria case for every 25 introduced cases (figure 1), but these averages are dominated by a few large outbreaks. Approximately 85 per cent of the estimates were less than 0.01 [36]. The estimated number of imported cases probably represents a conservative lower-limit estimate, since reported imported malaria cases are often estimated to represent less than half, and potentially only one-sixth of actual imported cases [37,38]. The estimated value of *R*_C_ therefore probably represents an upper-limit estimate since most individual cases in non-immune populations will present with fever, and since any detected malaria case triggers an investigation, chains of locally acquired secondary cases are much less likely to go undetected than isolated cases. Since all of these countries once sustained endemic transmission (i.e. *R*_0_ > 1), this analysis suggests that transmission potentials in malaria elimination countries have declined by a factor of at least 50. Elimination has become highly stable.

The bifurcating paths of elimination and endemic countries are a curious phenomenon not explained by current theory. Why should malaria elimination be a stable endpoint when the reduction of burden to even very low levels remains unstable? In one famous example, Sri Lanka reduced malaria to only six locally acquired cases, only to see incidence rebound to over 500 000 six years later [39]. Yet 46 countries have avoided any resurgence despite continued, and apparently increasing, importation over decades [40–42]. Hypotheses to explain this discrepancy fall into four overarching categories. These four sets of causes are not mutually exclusive, since each one could each explain some portion of the 50-fold decline in transmission. First, countries that successfully eliminated malaria may be different from those that were unsuccessful in terms of factors such as socioeconomics, vectors, climate or transmission intensity, and some of these factors may also explain the reductions in potential transmission post-elimination. According to this theory, the elimination effort revealed rather than caused differences between countries. Second, changes—whether caused by biology or by economic factors acting on the biology—may have occurred as a result of elimination that caused unexpected stability, potentially including increased economic growth that reduced baseline vectorial capacity or behavioural changes in the population. Third, elimination is stable due to some changes in the vector populations, such as local elimination. Fourth, successfully eliminating countries may not have been unusual either before or after elimination, but importation from abroad may be far less effective at rekindling transmission than transmission from residual foci in unsuccessful countries. In the following sections, we explore these hypotheses, expand the discussion of the theory of transmission and, within that theory, examine several mechanisms in order to help explain the bifurcating paths.

## 3. Is elimination stable because successful countries are unusual?

Elimination may simply appear stable because the countries that achieved it are fundamentally different from those that did not with respect to factors such as climate, wealth, vector species or transmission intensity. If such differences are the primary drivers of the observed stability, there is no conflict with current elimination theory and it is probable that today's poorer, higher endemic countries will find elimination unsustainable even if it can be achieved.

One potential difference often cited is climate, since countries that eliminated malaria tended to be at the northern or southern margins of malaria's range. Climatic factors, such as seasonality, may have made countries suitable for elimination [43], but possibly not climate change. Since warming is generally expected to increase transmission, except perhaps when it is already very hot [44], a warming trend is unlikely to explain the post-elimination reductions in *R*_0_ [30]. Changes in rainfall and its seasonal patterns could also cause some of these changes [45,46], but there is as yet no evidence that these changes are responsible for large declines in transmission across the geographical range of malaria. The apparent stability of elimination may also reflect the fact that only richer [47], more urbanized [48,49] countries achieved elimination in the first place. The GMEP also coincided with a period of rapid economic growth and development in many countries. If growth or urbanization caused certain countries to eliminate, it may have also helped those countries maintain elimination.

The mechanism through which wealth and urbanization influence transmission is not entirely clear and probably varies from place to place. One hypothesis is that malaria transmission may be affected by a change in the nutritional status of populations [50,51]. Another is that economic development causes land use changes that eliminate vector habitat including construction of dams for vector management purposes [52], paving roads, changing patterns of agriculture, changing hydrology and draining swamps [53,54]. Also relevant are changes in human behaviours and housing quality that are associated with the accumulation of wealth and that reduce contact between humans and vectors. Closing up or improving houses, either by closing eaves, installing windows or screens, installation of air conditioning, or installation of tin roofs can prevent or discourage mosquitoes from feeding on humans and thereby lower transmission [55–57]. Further, these human adaptations can select for evolutionary changes in the vector populations, especially declining anthropophagy. Hackett studied the natural disappearance of malaria from parts of Europe by comparing areas that still had malaria with those that did not. He found that areas without malaria had vector populations that tended to feed on cattle but not on humans, and he concluded that the populations had evolved [58]. Other large effects on transmission can arise because of underlying demographical changes in human populations. Humphreys [59] proposed that the mechanization of agriculture ended sharecropping in the USA, and that the resulting migration removed humans from rural areas that had the highest transmission. To put it another way, economic changes can bring about demographical changes, especially in poor populations, that move people away from highly malarious habitat resulting in reduced transmission.

In urban environments specifically, vector habitat is limited. It can be destroyed or made unsuitable for a variety of reasons, from deliberate manipulation for public health improvement to polluted larval sites, to building roads and buildings where mosquito habitat once existed [60]. Models suggest that areas with high human population density but limited mosquito density have a difficult time sustaining transmission because it is the number of mosquito bites per person that determines mosquito-borne disease transmission intensity, and high human population densities dilute transmission by mosquitoes [15]. Other advantages of living in areas with high population density are better infrastructure, better access to healthcare, democratization and local institutional support. Finally, malaria control is much easier to organize in areas with high population density, and it is perhaps more cost-effective because of lower operational costs and larger benefits per person. Regardless of the reasons, it is clear that increasing urbanization leads to declining malaria [48,49,60].

**Figure 2.**
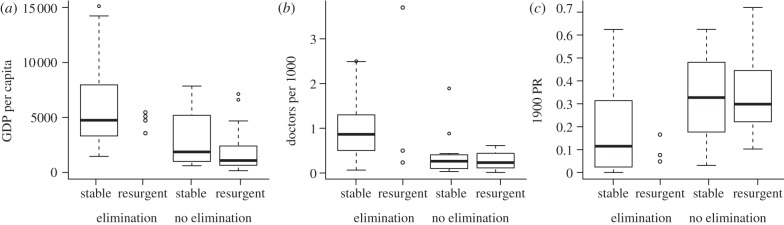
Boxplots comparing the properties of countries based on whether they achieved elimination or not and whether the endpoint was stable (no resurgence event) or some resurgence event. The properties compared were (*a*) GDP per capita (the *y*-axis was rescaled, which omitted five additional outliers from stable/elimination, ranging above $45 000); (*b*) the number of doctors per 1000 people; and (*c*) the parasite rate (PR) in 1900.

To restate the question, is there evidence that wealth and economic development could have reduced *R*_0_ in countries that successfully eliminated malaria to the point that resurgence risk was minimized? In addition to classifying country elimination programmes, Feachem *et al.* [34] provided comparative data on national wealth (purchasing power parity adjusted real per capita gross domestic product (GDP) in 2005 US dollars) and health system capacity (number of physicians per 1000 persons) at the time of the elimination. As expected, the 50 countries that eliminated successfully had higher GDP ($7598) and more doctors per capita (0.99) than countries that did not eliminate (figure 2*a*; GDP = $2069, *t* = −4.355, *p* < 0.001 and doctors = 0.31, *t* = −5.385, *p* < 0.001). They can also be compared with population-weighted prevalence rates from 1900 (Lysenko), which provide rough indications of the potential for transmission before vector control interventions had begun to be implemented [30]. Countries that eliminated had lower 1900 parasite rates (mean = 20.1%) than those that did not (figure 2*b*, mean = 33.3%, *t* = 3.077, *p* = 0.003).

An additional comparison can be made between those programmes that were found to have experienced resurgence events in a review by Cohen *et al.* [35] and those where no account of resurgence was identified. Countries that experienced resurgence, regardless of elimination success, had lower GDP (*t* = 4.236, *p* < 0.001) and fewer doctors per capita (*t* = 3.339, *p* = 0.001) than countries without resurgence. Additionally, countries that experienced resurgence had slightly, though not significantly, higher 1900 parasite rates (mean = 31.3%) than countries that did not experience resurgence (figure 2*c*; mean = 23.9%, *t* = −1.718, *p* = 0.090). However, none of these differences were significant when additionally controlling for whether or not the elimination programme was successful in a multivariate logistic regression model (for GDP, *z* = −1.769, *p* = 0.077; for doctors, *z* = 0.145, *p* = 0.884; and for 1900 parasite rate, *z* = −0.509, *p* = 0.611).

There were 14 countries that maintained elimination successfully despite having a per capita GDP that was lower than the poorest country that achieved but lost elimination (South Korea, with GDP = $3570). This analysis, while crude, suggests that countries that achieved elimination were indeed different from those that have been unsuccessful, but that these differences were only weakly predictive of which countries will experience resurgence once elimination is achieved. The stability of elimination requires additional explanation.

One additional consideration is that economic development and urbanization are not the only factors that could have produced large, unattributed changes in malaria transmission. In the second half of the nineteenth century, malaria started to decline in the United Kingdom [61], parts of mainland Europe [58], and the United States [62], and continued until malaria was eliminated. More recently, malaria transmission and endemicity have sharply declined in some areas of India [63], Mexico [18] and in some African cities [60]. While these declines are associated with increased wealth or urbanization, they are not the only putative causes. Recently, malaria vector populations in northern Tanzania, as reflected by vector catch data, have declined by 99 per cent for reasons that are not understood [64], and malaria was apparently declining in northern coastal Kenya before intervention coverage levels started to scale-up [65]. Random fluctuations and large trends in the potential for malaria transmission are, therefore, common. At least part of these changes could be classified as changes in baseline transmission (i.e. changing *R*_0_) that are maintained without further inputs.

## 4. Is elimination stable because of changes that occur as a result of its achievement?

Intensive malaria control programmes that aim to interrupt transmission tend to rely heavily on vector control. Most reasoning about the possibility for resurgence is based on the theory of vector control. Without permanent changes to the baseline (i.e. to *R*_0_), the vectors will retain their capability to transmit malaria—that is, vector control reduces vectorial capacity (i.e. from *V*_0_ to *V*_C_) as long as interventions are maintained, but it is assumed to naturally return back to the baseline (i.e. *V*_0_) after vector control has been relaxed. Unless there are changes in the sort of structural factors described above, *R*_0_ will remain high after control, and malaria resurgence is highly probable following the reintroduction of malaria. It is possible that elimination may lead to economic growth, which in turn may reduce *R*_0_ and help a country to maintain elimination once it is achieved [47]. However, direct evidence of such an effect is not strong [20,66]. Might other changes drive stability?

One aspect that current elimination theory has not considered is the transmission control effect of drugs, i.e*.* changes to *D*_C,0_. Antimalarial dugs are primarily regarded as a means of curing infections and reducing disease, but they can also reduce transmission. Though gametocytes are the life-stage transmitted to humans, they are relatively short-lived in blood compared with the self-propagating asexual stages [67]. The dominant effect of antimalarial drugs on transmission comes from curing these potentially long asexual infections, ending the production of new gametocytes, and thereby shortening the infectious period compared with untreated infections. Chemoprophylaxis and direct destruction of gametocytes are other secondary effects [68,69]. The quantitative reductions in transmission depend strongly upon ambient transmission intensity, health-seeking behaviour, drug policy and the infection and immune status of those being treated (see below). Existing models of controlling transmission with antimalarial drugs suggest that they could help to explain the low value of *R*_C_ observed in elimination settings, but not in endemic settings.

Most evidence also suggests that at high transmission intensity, antimalarial drugs have only a limited effect on controlling transmission [70–72]. The reasons are that most regimens do not directly affect mature gametocytes such that a person can remain infectious after being treated and, meanwhile, new infections can occur rapidly after treatment and re-establish gametocyte populations. Parasite transmission from recently treated individuals may be only temporarily depressed, and there are many other individuals who have not been recently treated. Drugs that do affect mature gametocytes, such as primaquine, reduce the reservoir of infectious parasites in those who have been recently treated [73], but even these reductions represent but a small fraction of the reservoir of parasites that remains in those who are carrying parasites asymptomatically [68]. The exception to this rule may be with well-designed mass drug administration programmes [74], which can temporarily reduce infectiousness for a large fraction of the population all at once, though even these may not work well in hyper- to holo-endemic settings [75,76]. An important feature of high intensity transmission is that part of the population is clinically immune, defined herein as a reduction in the relative risk of developing clinical symptoms of malaria from a new infection. Clinical immunity helps one to establish a stable reservoir of infection that is relatively unaffected by treatment with antimalarial drugs. Curiously, the persistence of malaria parasites in endemic regions may extend to areas with very low intensity, where there are several subpatent infections for every patent infection [77,78]. The cause of this pattern is as yet poorly understood [79].

At low transmission intensity and in elimination settings, however, drugs can have a much larger effect on transmission [69,80–82]. In these settings, the reservoir of parasites is relatively smaller, and clinical immunity is generally poorly developed, except possibly in transmission hotspots [83], so that most people who get malaria also get sick. A critical detail of *P. falciparum* biology is that most cases malaria infection in non-immune populations develop fever and other clinical symptoms on or around the 10th day of infection [84], though they may delay seeking treatment for a variety of reasons. *Plasmodium falciparum* gametocytes can be produced almost immediately, but they require approximately 12 days to mature before they are capable of infecting a mosquito [85]. Prompt effective treatment with drugs ends the asexual cycle, and depending on the drug regimen, it can also kill some developing gametocytes and substantially reduce net infectiousness of individuals [86]. *Plasmodium vivax* infections are different because of liver-stage hypnozoite infections that can relapse long after a primary infection [87], and because the gametocytes mature much more rapidly [86]. These two effects will produce slightly different patterns than *P. falciparum*, but treatment of infections will have similar properties. Infections that are promptly treated are infectious perhaps at a low level and for only a few days, but untreated or improperly treated infections are sporadically infectious over much longer periods, perhaps 6–8 months on average [77,88]. Simple logic thus suggests that prompt treatment of most new infections can sharply reduce transmission, but the quantitative effects are strongly dependent on the timing of treatment relative to the start of the infection. Mathematical modelling suggests that the transmission effect sizes grow extremely large as the fraction of new malaria infections treated approaches 100 per cent [69]. These effect sizes are highly sensitive to the fraction of new infections that goes untreated [69], so the contrasts are drawn even more sharply in low intensity versus elimination settings.

**Figure 3.**
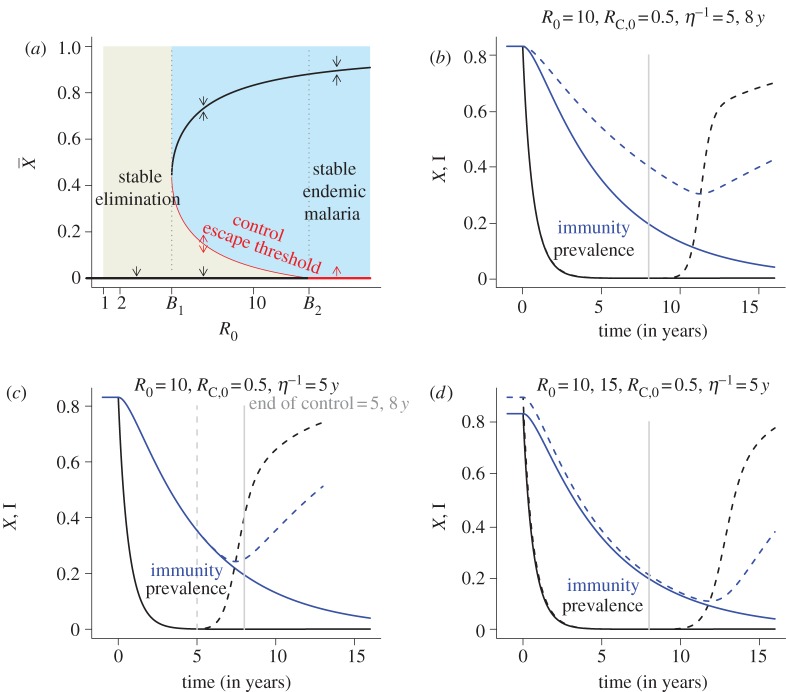
(*a*) Stickiness can be described rigorously in dynamic models where the fraction of incident infections that are treated and cured declines with the level of endemicity (i.e. 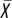). In this context, ‘sticky’ means that two stable steady states exist. This bifurcation diagram shows the steady state endemicity as a function of the intrinsic potential for transmission, described by the basic reproductive number *R*_0_. The stability of endemic malaria is strongly dependent on *R*_0_, and the stability changes at ‘bifurcation’ points called *B*_1_ and *B*_2_ (vertical dotted lines). For values of *R*_0_ below *B*_2_, malaria elimination is stable (thick black line at 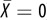). For values of *R*_0_ above *B*_1_, endemic malaria is stable (described by the thick black curve). If *B_1_* < *R_0_* < *B_2_*, then malaria elimination and endemic malaria are both stable, and elimination is ‘sticky.’ To achieve elimination when it is sticky, endemic malaria must be suppressed below a curve describing an unsteady state called a ‘control escape threshold’ (the thin red curve) long enough for immunity to wane. Details and analysis of the model are found in the electronic supplementary material. (*b–d*) To illustrate how the ideas in this diagram play out in a simulated vector control campaign, vectorial capacity was sharply reduced for a time (at *t* = 0) and then allowed to return to its baseline (the vertical grey lines). If vectorial capacity is suppressed for long enough, then immunity wanes and elimination is a stable endpoint. If control is relaxed prematurely, then malaria is resurgent. (*b*) Two different scenarios were simulated with different rates of waning immunity (i.e. 5 versus 8 years). The outcome is sensitive to the rate at which immunity wanes—the slower it wanes, the longer vector control must be sustained for elimination to become sticky. (*c*) Two different scenarios were simulated to show the effect of the duration of sustained control (i.e. 5 versus 8 years). For a fixed rate of waning immunity (i.e. 5 years), the long-term outcomes depend on the duration of the control programmes—vector control must be sustained long enough for malaria immunity to wane, and if it is not, then malaria will be resurgent. (*d*) Two different scenarios were simulated to show the effect of baseline. All else equal, the outcome is sensitive to the level of baseline transmission—the higher the baseline, the longer vector control must be sustained before immunity has waned sufficiently.

These treatment effects produce complex dynamics when they are put into simple transmission models. Dynamic feedbacks between immunity, transmission and health-seeking behaviour can create a situation where there are two stable equilibria, and when considered with respect to *R*_0_, this can create a backwards bifurcation in malaria dynamics [89,90]. This has been illustrated heuristically here with a Ross–Macdonald model in which the fraction treated is dynamically dependent on the level of immunity, which tracks endemicity (figure 3, and in the electronic supplementary material). The key features of the dynamic behaviour in this model are characterized by two critical values of *R*_0_, called bifurcation points, at which the dynamic behaviour of the system changes qualitatively. Below the first bifurcation point (called *B*_1_), elimination is stable and there is no endemic malaria. Above the second bifurcation point (called *B*_2_), endemicity is highly stable while elimination is not.

Between the two bifurcation points, malaria elimination and endemic malaria are both stable. This leads to a kind of path dependency: the same place (as defined by its *R*_0_ value) could have stable endemic malaria or stable elimination, depending only on the immunity in the population acquired from its recent history with malaria. In such places, malaria elimination must be achieved through vector control or through some other means and sustained long enough for clinical immunity to wane (figure 3*b,c*). This suggests that it may not be enough to eliminate the reservoir of parasites, but that it may also be necessary to continue to apply these interventions through a transition period. After clinical immunity has waned, it may be possible to scale back vector-based interventions without incurring a dramatically increased risk of resurgence—to a point where the control effects of vector control alone reduce transmission to below the higher bifurcation point (i.e. where *R*_0_ < *B*_2_, figure 2*d*). This theory suggests that treatment of symptomatic patients with drugs can play a dominant role in achieving and maintaining elimination only in places where *R*_0_ < *B*_1_. In places where *B*_1_ < *R*_0_ < *B*_2_, drugs will not play an effective role in achieving elimination, but they can play a major role in maintaining elimination after it has been achieved. In places where *R*_0_ > *B*_2_, elimination must be achieved through intensive vector control and then some level of vector control must be maintained to continue to suppress *R*_C_ after elimination to remain malaria-free.

Another important feature of the dynamics is a threshold on endemicity at which stable elimination escapes control so that endemic malaria transmission is re-established. In mathematical terms, it is an unstable manifold connecting the stable endemic manifold at *B*_2_ and the stable elimination manifold at *B*_1_ (figure 3*a*). Crossing this threshold changes transmission from tending towards stable endemic malaria to tending towards stable elimination or vice versa. The height of the control escape threshold is thus of interest, as is the rate at which immunity waxes and wanes. In general, immunity is expected to change more slowly than endemicity, and it should reflect the recent history of endemicity. As immunity adjusts, elimination should become more stable as immunity wanes after the interruption of transmission. Similarly, clinical immunity can develop during an outbreak and eventually create conditions that pull back towards re-establishment of endemic transmission. The lesson from the heuristic model is that crossing the control threshold changes a place from stable endemic to stable elimination, but the shape of this manifold depends on the rate that immunity waxes during an outbreak, or wanes after a period of control (figure 3*b–d*). The ability to control an outbreak is expected to decline as population immunity builds, but the possibility of control remains high because clinical protection tends to develop only after multiple exposures. On the other hand, the rate that clinical immunity wanes is poorly understood.

In elimination settings, reported malaria cases and routine medical attention are often complemented by outbreak response protocols that include taking patient histories and reactive case detection [7]. As new cases are generated, they provide information about the cases that were not promptly treated, which may then be detected and truncated. During an outbreak, enhanced vigilance makes introduced cases (i.e. those infected by the index case) more likely to be detected and treated. The biology of malaria also facilitates outbreak responses, at least operationally, because in excess of two weeks are required before a human infected with *P. falciparum* produces mature gametocytes, and approximately two weeks more are required for pathogen development in mosquitoes. This month-long gap provides time to mount an outbreak response following the detection of an imported case, though the operational challenges of promptly detecting and responding to an unfolding outbreak may be very much greater for some very large countries and relatively inaccessible regions. Outbreak response increases the value of *B*_2_ far higher than is possible with routine medical attention. After clinical immunity has waned, malaria (at least, where *R*_0_ is below *B_2_*) is like other diseases that are highly amenable to outbreak control [91].

The value of *B*_1_, the level at which malaria can be controlled only with drugs, will depend on many additional aspects of the vector biology, parasite infections, human behaviour, health systems, access to healthcare, seasonality and immunity that cannot be evaluated with these simple, heuristic models. Importantly, this breakpoint is made apparent by elimination. In areas where malaria remains endemic, the relevant policy question is the value of *B*_2_, the highest value of *R*_0_ at which malaria would remain stable following elimination because of factors related only to the health system. This is of great importance for strategic planning for elimination because it tells countries how much (or alternatively where) they can relax vector control (thereby saving money) following elimination without incurring a major risk of resurgence. The higher *R*_0_, the higher the fraction of cases that must be treated promptly to suppress transmission, or the more efficient the outbreak response required. The breakpoints from this simple model should be regarded only as a heuristic. Biological and other limits on the ability to treat every case or mount a perfect outbreak response suggest that stickiness occurs for low to moderate values of baseline transmission, *R*_0_, but that it is much higher to maintain for high transmission settings.

In the simple heuristic model described herein, the breakpoint *B*_2_ depends mainly on the fraction of infections that would be promptly and effectively treated in the absence of any clinical immunity. All else equal, the likelihood of symptoms is expected to increase with decreasing immunity, but there is a large degree of variability in the presentation of malaria, even in non-immune populations [92]. One biological constraint is a well-documented but poorly understood pattern of malaria infections that may not produce any symptoms or that are only mildly pathogenic [79]. If a low-pathogenicity parasite phenotype were stable across individual human infections, it would be the most difficult to exclude from a population, though even a few symptomatic cases would trigger an outbreak response that could lead to detection of the whole cluster. With outbreak control, depending on the efficiency of reactive case detection in the response, even pathogens of low pathogenicity would eventually be detected and eliminated.

Subject to the biological limits, in moderate-to-low transmission settings the development of wealth can help to reinforce health-seeking behaviour so that a higher fraction of cases is treated. While it may seem impossible to treat a high fraction of malaria infections in malaria endemic areas today, it does not seem implausible to do so in areas where there is no locally endemic malaria. What accounts for the difference in perception? In addition to the health systems, the combination of increased wealth and changing social norms after a long absence of malaria can also improve health-seeking behaviour for malaria. In malaria endemic areas, malaria infections may be tolerated because infections occur with high frequency, with mortality being perceived as a rare but expected possible outcome of infection, and so malaria may be regarded as ‘normal’. The relatively high levels of clinical immunity acquired by adults can further reinforce these perceptions. After elimination, with waning immunity and no recent experience with malaria, social norms can change so that malaria infections become less tolerable. After immunity has waned, wealth increased, health systems improved and social norms changed, a much higher fraction of incident malaria infections could be seen and treated promptly and properly, assuming sufficient health system capacity. Thus, changes in health-seeking behavioural norms for malaria could help explain why malaria is difficult to get rid of in the first place, but much easier to keep it out once it is gone.

An open question is whether ‘stickiness’ is a robust property of more realistic malaria transmission models, whether it could be a realistic explanation for the observed stability of elimination, and where stickiness could be realistically applied elsewhere. Stickiness in this model is, in fact, highly sensitive to the fraction of incident malaria infections that present with clinical symptoms, and it would almost certainly be important in other models. More realistic models can, however, include spatial dynamics and heterogeneous biting, various manifestations of immunity all with potentially different rates of waxing and waning, and most of all, suites of interventions and protocols that reflect country policies. Some preliminary simulations suggest that stickiness is a robust property of models, and that the most important factors affecting the range of parameters over which elimination in a country can become sticky may be under the influence of the health system, especially the efficiency of control achieved during outbreak responses. This question will be addressed in future work.

## 5. Is elimination stable because of factors related to vectors?

Differences among vector species and the composition of vector species present in an area could explain some part of stickiness. We have described how *R*_0_ plays an important role as a threshold parameter in determining whether malaria progresses towards extinction or towards an endemic state, and how *R*_0_ can be decomposed into two factors, representing the human and vectorial components of transmission. The vectorial component, *V*_0_, is determined in part by the ratio of mosquitoes to humans, the proportion of mosquitoes feeding on humans, the lifespan of mosquitoes and the duration of sporozontal development [93]. There is immense variability in these parameters and in the potential of different mosquito species to transmit malaria [94], and therefore in the intensity of transmission under contemporary conditions [95]. In some parts of sub-Saharan Africa, where baseline transmission reaches its highest measured intensity, eliminating malaria with current tools will prove to be an enormous challenge [96,97], and it seems highly improbable that it would be possible to maintain elimination without enormous ongoing investments in vector control [18].

All of the above complications will affect the ability of control measures to reduce the transmission of malaria through vectors, but the underlying model assumes that vector control changes *V*_C_, but not *V*_0_. Once the effects of vector-based interventions decayed or were removed, the mosquito populations would rebound and transmission by mosquitoes would resume at a level determined by the pre-control baseline. One way of explaining stickiness is that vector-based interventions cause changes to the baseline. As previously mentioned, it is possible that mosquito behaviour could change, either through evolution, such as the changing patterns of anthropophagy that Hackett associated with declining malaria in Europe [58], or that there could be some permanent changes in ecology through changes in land use. The question is whether these sorts of permanent changes can come about because of control.

Because control efforts such as bed nets and spraying might differentially impact various vector species in an area, the relative proportion of vectors may change (i.e. the proportions of indoor to outdoor biting mosquitoes, for example). These sorts of changes are not typically considered a change in baseline, but in some cases they have been associated with durable changes in the ecology of the vector species that transmit malaria. One example is South Africa, where the use of DDT eliminated the vector *Anopheles funestus* from much of its former range [98], leaving some isolated pockets of *An. funestus* as well as small populations of *Anopheles arabiensis*. When the policy shifted from using DDT to pyrethroids, *An. funestus* remained rare or absent for a time, but then pyethroid-resistant *An. funestus* eventually returned along with resurgent transmission. Notably, resistance to the dominant drug was another possible factor in the resurgence. A shift back to DDT and a change in drug policy saw the malaria cases fall once more [99]. This example suggests that it is possible, in theory at least, for vector control to bring about the local elimination of one or more of the vectors.

There are several potential mechanisms by which elimination of one vector from its local habitat brings about more durable changes in vector ecology. One is competitive exclusion, in which the eliminated species may not be able to reinvade its old habitat given that carrying capacities have been filled by other species, such that a temporary control effort would permanently reduce vectorial capacity [100–102]. Another possible mechanism is that some positive feedback between adult vectors and aquatic habitat is required—such as adult ‘transmission’ of mutualistic microbes among aquatic habitats [103,104]. Indeed, depending on the mode of control, these mechanisms might increase transmission if the invading species have higher vectorial capacity than the original mix of vector species. These nonlinear effects of vector control, a kind of stickiness in mosquito populations, could explain persistent declines in transmission. Further, it is also possible that vector control efforts may change the biting patterns of vectors, such as the declining anthropophagy in Europe [58]. These nonlinearities may also decrease or increase the effectiveness of control efforts beyond the effect sizes predicted by models of vectorial control that do not incorporate these phenomena.

## 6. Is elimination stable because importation is not effective at rekindling transmission?

The low estimated reproductive numbers in malaria elimination countries reflects potential malaria transmission in the largely urban places where the cases were imported. The potential for transmission may be much higher in the more remote parts of the country that rarely import malaria. Urban areas tend to be travel hubs and frequent travel destinations, but they also tend to have the lowest levels of malaria endemicity [49]. A recent study showed that Nairobi was the most common destination for intra-national travel in Kenya [105]. More importantly, travel among small rural communities was comparatively rare, even over short distances with small populations. Remote areas with high potential for transmission are usually the last to eliminate malaria, but they are neither frequent travel destinations nor home to frequent travellers. This raises the interesting questions about how human mobility patterns reveal or obscure the underlying potential for transmission, and how those patterns reinforce the overall stability of malaria elimination.

**Figure 4.**
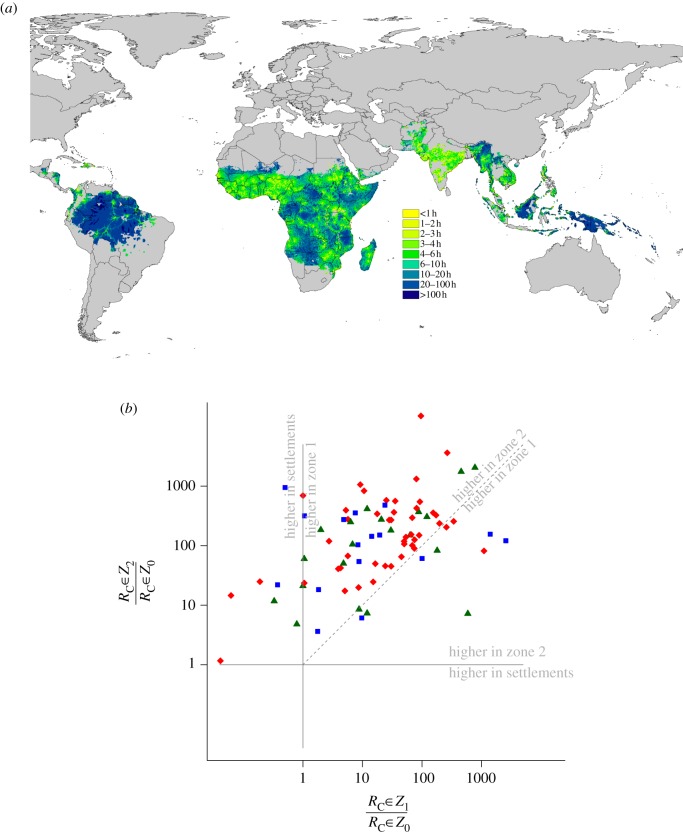
(*a*) Estimated travel time to the nearest settlement of population more than 100 000 [106] within the contemporary limits of *P. falciparum* transmission [95]. (*b*) Scatterplot comparing mean ratios of *R*_C_ values for settlements (zone 0 or *Z*_0_) compared with areas in zone 1 outside of settlements but less than 4 h travel time from a settlement (*Z_1_*), and compared with areas in zone 2 greater than 4 h travel time from settlements (*Z*_2_). Each dot represents a country and blue squares are for the Americas, red diamonds for Africa and dark green triangles for Asia. Two one-to-one comparison lines are added and annotated to show regions of the graph where transmission is higher in settlements than either zone 1 or zone 2. Above the diagonal dashed line, transmission is higher in zone 2 than zone 1. The general pattern is that *R*_C_ values tend to be in the upper triangle: lowest in settlements, and highest more than 4 h travel time from settlements. Those in the upper triangle have the greatest potential for ‘stickiness’.

To explore this hypothesis, we examined the contemporary situation of whether remote locations that probably see relatively little incoming and outgoing travel exhibit higher transmission than urban areas, which tend to be significant travel destinations. A recently constructed global map of *P. falciparum R*_C_ [95] was compared with a map of remoteness, measured by estimated travel time to the nearest settlement of population size more than 100 000 [106], shown in figure 4*a*. For each endemic country, a consistent relationship was seen of substantially higher *R*_C_ values as remoteness increased. For over half the countries, the mean *R*_C_ values seen in areas greater than four hours travel time away from the nearest settlement were more than 100 times than those for urban areas (figure 4*b*).

These patterns suggest that the areas with the highest potential for transmission will be more remote and will therefore tend to have the lowest rates of malaria importation. Areas that are frequently visited will have lower *R*_C_ values and better health systems, which will tend to reinforce the stickiness of malaria elimination. As malaria control efforts work towards elimination, malaria will tend to contract into these remote hotspots [83], places with the highest levels of transmission. After the last parasites have been eliminated from the remaining foci, transmission cannot start again until a malaria parasite is reintroduced. The absence of malaria is reinforced by changes in attitudes to malaria, changing immunity and improvements to the health system, for the reasons discussed in the preceding section. As elimination proceeds, fewer and fewer highly endemic foci will remain, and these areas will probably be more remote and isolated from one another since areas with highest transmission today tend to be located in more rural regions (figure 4).

What is likely to remain even after a large change in *R*_0_ is a landscape with a highly heterogeneous potential for transmission but no malaria, what has been called ‘anophelism without malaria [107]’. This theory would predict, for example, that there are places in malaria elimination countries where malaria would be highly explosive if it were introduced today. Indeed, there have been a few highly explosive epidemics in malaria-free countries, the most recent occurring in Greece [108]. The aggregated data describing importations, however, demonstrate that these large outbreaks are the exception to the rule. On the other hand, endemic transmission in remaining hotspots is, by definition, a stable reservoir that threatens to reintroduce malaria to surrounding areas. If low endemic malaria is maintained by health systems and treatment, then there is also a potential for explosive and deadly epidemics if malaria spreads out of these foci and escapes control, such as occurred in Madagascar in 1986–1988 [21]. This would suggest that elimination, far from having no benefits, separates malaria from the areas of highest risk and brings about a sharp reduction in the risk of future epidemics.

Collectively, these examples suggest that one of the main benefits of eliminating malaria is to minimize the risk of reintroducing malaria into one of the remaining hotspots. This is not to suggest that imported malaria would not continue to pose real risks, but that some sorts of travel pose greater risks than others. In particular, as the distances increase between areas where malaria is transmitted and areas where it could potentially be transmitted, importation is associated with shifts in the dominant modes of travel, in the type of traveller and in the destination. Overland travel over short distances would pose the greatest risk, but as the distance to malaria increases, travel patterns will tend to reinforce the stability of elimination.

## 7. Discussion

Malaria elimination has been a highly stable endpoint for those countries that reached this goal. New analysis of the evidence suggests that it has become highly dynamically stable: malaria transmission has declined by at least 98 per cent (i.e. 50-fold) in these countries since malaria was endemic [36]. Endemic malaria has also been highly stable. This bifurcating pattern would seem to contradict the standard theory of malaria transmission and vector control. If malaria elimination is in fact a highly stable state, then pursuing it may not be the endlessly costly, risky strategy that many believe. This finding would be significant for countries contemplating elimination and for endgame planning for malaria eradication, but the appropriate application depends on the cause of the stability of elimination. The critical question is whether economic development or random fluctuations caused large declines in malaria transmission, or whether changes were in fact caused by malaria elimination. If malaria elimination requires a spontaneous decline in transmission brought on by economic growth or urbanization, then programmes should aim to minimize the burden of malaria until transmission spontaneously declines. If, instead, elimination causes economic development and stability to reduce transmission by vectors, or if it brings biological and sociological changes that enhance the effect sizes associated with antimalarial drugs so that imported cases have very low probabilities of restarting endemic transmission, then elimination is a highly desirable endpoint, and it should be aggressively pursued and supported.

Our analysis suggests that it would be unwise to conclude that the same factors explain the patterns in every country, or that elimination will reliably become sticky in every country. Elimination appears to have been caused by economic development in some cases, such as the USA, but in many other cases, elimination appears to be the result of GMEP era malaria elimination programmes in countries. It has been proposed that malaria causes a poverty trap by lowering economic productivity [47,109]. It is possible that the GMEP-era programmes helped break health-related poverty traps and made it possible for economic growth to occur, thereby further reducing transmission [20,66]. Further, it is also possible that trends in economic development encouraged changes that then reinforced malaria elimination (for example, screen door usage, improved sanitation conditions, etc.). The interplay of economic growth brought on by malaria reductions and economic growth for other reasons both operated together to reinforce malaria elimination in certain countries. Because these two forces are intertwined, it is difficult to identify whether malaria reductions caused economic growth, whether growth caused reductions, or both at the same time, but the likelihood is that both factors work together in different countries at different times.

Indeed, in some countries, direct estimates of vectorial capacity in places without malaria confirm that the vectors are still present and fully capable of transmitting malaria, though in other countries the vectors evolved or went locally extinct. Most of the *R*_C_ estimates exceeding 0.5 were associated with a few large outbreaks spanning several years in Armenia, Greece, Mauritius and Turkmenistan. The recent outbreaks of malaria in Greece [110], and a few clusters of introduced cases rapidly staunched in Singapore [111], demonstrate that the vectors are still present in at least some places. These examples illustrate that there are still places where explosive epidemics could occur even in countries where malaria has effectively been eliminated. The theory introduced here suggests that control must be sustained through a transition period before elimination becomes sticky, and while other factors were associated with elimination success, the countries that eliminated were also among the earliest to initiate elimination programmes. An examination of all these cases suggests that malaria elimination becomes sticky for different reasons in different countries, and that there is a role for a careful assessment of the feasibility of malaria elimination on a country-to-country basis.

If it is true that malaria elimination is stable because of changes that occur after it is achieved, then malaria eradication is not at all like smallpox eradication, and the lessons that have been drawn from the sole successful human disease eradication programme should be applied cautiously to malaria. The end of routine smallpox vaccination saved approximately $450 for every dollar invested [112], but vaccination programmes needed to be maintained until the last case of smallpox resolved. Prematurely ending vaccination would have led to loss of ‘herd immunity’ and unacceptable risks. Thus, the economic benefits of smallpox eradication were only realized after eradication. To put it another way, if there had not been a last case of smallpox, then there would still be an imminent threat of a smallpox epidemic, and all national smallpox vaccination programmes would have been maintained. Once the last case was gone, the threat disappeared. Smallpox eradication was, therefore, an all-or-nothing affair.

One of the lessons that this analysis calls into question was summarized by Soper, who said ‘there is no such thing as a partial success in species eradication; one either achieves glorious success or dismal failure [113].’ All the evidence suggests that the GMEP was a partial success. The bifurcating history of countries that have achieved elimination and the sharp declines in potential transmission in elimination countries suggest that malaria is not an all-or-nothing phenomenon. The dynamics of the malaria eradication endgame are much less risky than smallpox for several reasons. Vaccination was the way to reduce smallpox transmission, but malaria transmission can be reduced in several different ways, which makes the sustainability of malaria elimination more robust. Theory suggests that the ability to control malaria through one of these modes—routine treatment of clinical malaria—increases as immunity wanes. After elimination, the potential for control is further enhanced through outbreak control. Together, routine treatment of malaria and outbreak control can stop malaria from re-establishing transmission in most places. It may thus be possible to abandon vertical malaria programmes after achieving elimination in areas with suitably strong health systems, which makes it possible to relax mass distribution of vector control and recoup enormous savings. This may not be true everywhere, and this theory of control by drugs could provide a basis for making those decisions.

The theory presented here for antimalarial drugs and health systems suggests that malaria immunity works in a fundamentally different way from immunity to acute immunizing infections. Herd immunity to smallpox, measles, polio and other acute and immunizing infections protects communities, but it would wane if vaccination were relaxed. This sort of immunity is clearly a public good. With malaria, however, the effects of clinical immunity present a more complicated picture. Clinical immunity to malaria is a private good in protecting against disease, i.e. it lowers an individual's chance of severe morbidity and/or mortality. Clinical immunity also creates a negative externality with respect to control by reducing the capacity to treat malaria through the health system. To eliminate malaria then, transmission must be suppressed long enough for clinical immunity to wane, else the risk of re-establishing endemic transmission is much higher, but individuals must also be assured of access to drugs because of the increased risks of complications from infection. The processes that give rise to breakpoints should be identified and tested as they play an important part in elimination and the eradicability of malaria [114].

This theory makes several recommendations for elimination and eradication. First, assuming elimination's stability is to an important degree a result of elimination and is not dependent only on preceding structural requirements such as economic development, climatic factors or ecological factors, as the available evidence suggests, then elimination is a highly desirable endpoint that should be supported by international organizations. Second, strengthening health systems is an essential part of malaria elimination since they will increase the fraction of infections that are rapidly treated. Low and intermediate *R*_0_ regions with strong health systems can move towards elimination with a reasonable expectation of remaining malaria-free, and other countries can prepare for elimination through a combination of vertical malaria programmes and health system strengthening. Third, since the risks of an outbreak increase with each imported case—and rise particularly quickly with each imported case that finds its way to a receptive region—the costs of remaining malaria-free and the risks of re-establishing endemic transmission increase with the number of imported cases. Conversely, each country that eliminates malaria exports less malaria, creating a public good for its neighbours. The most cost-effective strategy may be elimination of malaria from countries that belong to well-connected regions. A critical question is the spatial scales at which malaria elimination becomes sticky. Can elimination become self-reinforcing in some sub-region of a country? If so, then it would facilitate the success of, for example, the staged elimination campaign in Indonesia [115,116] and in the Philippines. As the size of a malaria-free region increases, malaria importation in the centre of the region decreases, and this further reinforces the stability of elimination.

Finally, the expense of maintaining an adequate outbreak response capacity, which is largely borne by countries, could be supplemented by an international resource to provide a kind of ‘insurance’. There may be a small but real risk of large, catastrophic epidemics in remote areas of some elimination countries if it should escape control [21], despite the apparent stability of elimination. Well-funded and well-trained international malaria outbreak response teams could prevent large and catastrophic outbreaks. This would guarantee the stability of elimination and would also provide an incentive for countries to invest in elimination. Historical precedents for such an idea do exist in the Rockefeller Foundation activities during the first half of the century [117].

It is highly improbable at present that all countries could pursue elimination with a high degree of success. Countries are at different stages of preparedness, with different baseline levels of transmission, operational challenges and financial constraints [23]. Some part of the technical challenge of elimination involves malaria importation, and is a dynamically changing threat. It is possible that, as neighbours eliminate malaria, the incentives to achieve malaria elimination also change [118]. Maps of malaria [95,119], estimates of importation [120,121], improved understanding of transmission and assessments of the feasibility of elimination [7,19] should all be a routine part of strategic planning. With such planning, the geographical range of malaria can be deliberately shrunk, and this strategy can be applied like a ratchet, progressing region by region, until malaria has been eradicated.

## 8. Conclusions

The permanent contraction in the geographical range of malaria achieved during the GMEP, and its subsequent abrupt end created a natural experiment. Among the most relevant outcomes was the high degree of stability of elimination at the level of a country. The reasons for this stability are of great interest. Systematic analysis of the available data suggests that while structural factors such wealth may play an important role in this stability, there are good reasons to believe elimination also reinforces its own stability through a combination of post-elimination improvements in case detection and treatment, changing travel patterns and potentially economic development. The retrospective analysis presented here has intrinsic limitations, but the question is of sufficient interest that some further research could be devoted to exploring these ideas. The theory presented here suggests the path to elimination differs for countries, depending on their baseline transmission and their neighbours. Stickiness does not present a one-size-fits-all solution. Elimination must be achieved through a combination of health system strengthening and through intensive vector control sustained long enough for malaria immunity to wane. Elimination is a highly desirable goal for countries, and may be a worthy goal regardless. The costs of sustaining elimination may be much lower than projected for countries with well-developed health systems and with low-to-moderate baseline transmission potential. Elimination is also likely to be stable for isolated countries even if they have reasonably high potential for transmission, but future work will be necessary to define the crucial thresholds for this stability. International progress towards elimination could be encouraged through regional coordination and through development of an international resource to control post-elimination outbreaks and reinforce the stability of elimination. For malaria, at least, eradication is not all-or-nothing. It may be achievable through country elimination programmes that achieve partial local successes. Viewed strategically, this defines a pattern of spatially progressive elimination as each country elimination programme moves the ratchet one notch towards global eradication.

## Supplementary Material

Online Electronic Supplement
