# Supplementary material for: A sticky situation: the unexpected stability of malaria elimination
Source: Philos Trans R Soc Lond B Biol Sci. 2013 Aug 5;368(1623):20120145. doi: 10.1098/rstb.2012.0145 (PMC3720043; doi:10.1098/rstb.2012.0145)
Supplement: Online Electronic Supplement [file rstb20120145supp1.pdf]

Phil. Trans. Roy. Soc. B **368** doi: 10.1098/rstb.2012.0145

Online Electronic Supplement:

A Sticky Situation: the Unexpected Stability of Malaria Elimination

David L Smith[1,2,3,4], Christinah Chiyaka [5], Justin M Cohen [6], Geoffrey Johnston [1,2], Peter W. Gething [7], Ramanan Laxminarayan [3], Simon I Hay [4,7], Andrew J Tatem [4,5,8]

1. Department of Epidemiology, Johns Hopkins Bloomberg School of Public Health, Baltimore, MD
2. Malaria Research Institute, Johns Hopkins Bloomberg School of Public Health, Baltimore, MD Emerging Pathogens Institute, University of Florida, Gainesville, FL
3. Center for Disease Dynamics, Economics and Policy, Washington, DC
4. Fogarty International Center, NIH, Bethesda, MD
5. Emerging Pathogens Institute, University of Florida, Gainesville, FL
6. Clinton Health Access Initiative, Boston, MA
7. Spatial Ecology, Department of Zoology, Oxford University
8. Department of Geography, University of Florida, Gainesville, FL

A Heuristic Model for Control with Antimalarial Drugs:

We let V denote vectorial capacity (1, 2) extended to include net transmission efficiency. Using notation from Smith & McKenzie (3), it is:

$$V = \frac{ma^2bc}{g}e^{-gn}.$$

Following the tradition of models by Ross (4), and later Macdonald (5), we minimize the role of mosquitoes to determining its intensity (6). We also make a further simplifying assumption that transmission rates are quite low, such that it is sensible to make an approximation about the equilibrium sporozoite rate:

$$\frac{acX}{g + acX}e^{-gn} \approx \frac{a}{g}e^{-gn}cX.$$

This allows us to write a highly simplified model for transmission by mosquitoes:

$$\dot{X} = VX(1 - X) - rX.$$

In this equation, $R_0 = V/r$, and if $R_0 > 1$, then malaria will be endemic with equilibrium prevalence is $\bar{X} = 1 - 1/R_0$.

We make the simplifying assumption (useful for the purposes of developing a heuristic model) that a person is either treated promptly and immediately with a drug thereby ending all infectiousness, or that the person is untreated until the infection resolves. The fraction of individuals that is treated is assumed to depend on the level of clinical immunity in the population, which is assumed to track prevalence but perhaps with a lag:

$$\dot{I} = \eta(X - I).$$

Finally, we assume that the fraction of the population that is promptly treated is a linear function of the level of immunity:

$$\rho_I = P - (P - Q)I.$$

The fraction treated with no immunity is P, but with maximum immunity, it is Q.

Treatment modifies the dynamics of malaria, as described by the equation:

$$\dot{X} = VX(1 - \rho_I)(1 - X) - rX.$$

At the equilibrium, $\bar{X} = \bar{I}$, and it is possible to solve for the equilibrium prevalence as a function of four parameters:

$$\bar{X} = \frac{-(2P - 1 - Q) \pm \sqrt{(2P - 1 - Q)^2 - 4(Q - P)(1 - P - R_0^{-1})}}{2(Q - P)}.$$

The first bifurcation point of this model, denoted $B_1$is given by setting the radical to zero:

$$R_0 = \frac{4(P - Q)}{(1 - Q)^2}$$

The second bifurcation point, $B_2$is given by:

$$R_0 > \frac{1}{1 - P}.$$

Elimination & Resurgence:

A table was compiled based on Table 2.6 from Feachem *et al.* (7) and compared with resurgence data from Cohen *et al.* (8), and with digitized malaria prevalence from the Lysenko map following Gething *et al.* (9). These are plotted, stratified by whether a country achieved elimination or not and had a resurgence or not for adjusted GDP per capita (Fig 3a), doctors per 1,000 population (Fig 3b), and the average parasite rate for the country in 1900 (Fig 3c).

References

1. Macdonald G. The analysis of the sporozoite rate. Trop Dis Bull. 1952;49(6):569-86. Epub 1952/06/01.
2. Garrett-Jones C. The Human Blood Index of Malaria Vectors in Relation to Epidemiological Assessment. Bull World Health Organ. 1964;30:241-61. Epub 1964/01/01.
3. Smith DL, McKenzie FE. Statics and dynamics of malaria infection in Anopheles mosquitoes. Malar J. 2004;3:e13. Epub 2004/06/08.
4. Ross R. Report on the prevention of Malaria in Mauritius. London: Waterlow and Sons Limited; 1908.
5. Macdonald G. The analysis of infection rates in diseases in which superinfection occurs. Trop Dis Bull. 1950;47(10):907-15. Epub 1950/10/01.
6. Smith DL, Battle KE, Hay SI, Barker CM, Scott TW, McKenzie FE. Ross, macdonald, and a theory for the dynamics and control of mosquito-transmitted pathogens. PLoS Pathog. 2012;8(4):e1002588. Epub 2012/04/13.
7. Feachem RG, Phillips AA, Hwang J, Cotter C, Wielgosz B, Greenwood BM, et al. Shrinking the malaria map: progress and prospects. Lancet. 2010;376(9752):1566-78. Epub 2010/11/03.
8. Cohen JM, Smith DL, Cotter C, Ward A, Yamey G, Sabot OJ, et al. Malaria resurgence: a systematic review and assessment of its causes. Malar J. 2012;11(1):122. Epub 2012/04/26.
9. Gething PW, Smith DL, Patil AP, Tatem AJ, Snow RW, Hay SI. Climate change and the global malaria recession. Nature. 2010;465(7296):342-5. Epub 2010/05/21.
